# ATP consumption in molecular signaling of CA1 Hippocampus neurons

**DOI:** 10.1186/1471-2202-16-S1-P56

**Published:** 2015-12-18

**Authors:** Nikon Rasumov, Erik De Schutter

**Affiliations:** 1 Okinawa Institute of Science and Technology Graduate University, Onna, Okinawa 904 0495, Japan

## 

The human brain consumes 10^6 ^times less energy than the currently fastest super computer [1], while maintaining a comparable performance in many demanding tasks [2]. This energetic efficiency has been suggested to result from primitive computations on a molecular level [3]. However, while the importance of ion channels on energy efficiency has been the primary focus of research [4,5], most computations occur at the molecular level prior to the amplification step and prior to the information transmission through neurons. We calculate the amount of energy consumed by such computations and compare their structural and functional properties. As a starting point, we chose 2000 reactions in the signaling pathways of CA1 hippocampal neurons [6]. As not every reaction consumes either one or zero ATPs, we undergo a wide literature search to identify the exact energy consumption of over 60 million of possible feedback loops. We find that the number of ATPs consumed is related with size of positive feedback loop. Hence, this study provides the first systematic and detailed attempt to investigate the energy consumption of information-storing primitive computations and points towards energy efficient motifs for synthetic biology.
